# Immune-related adverse events are associated with favorable clinical outcomes in esophageal cancer treated with immune checkpoint inhibitors: a systematic review and meta-analysis

**DOI:** 10.3389/fonc.2026.1948772

**Published:** 2026-10-06

**Authors:** Jiajia Fan, Dongmei Chen, Liqun Jia

**Affiliations:** 1 Graduate School, Beijing University of Chinese Medicine, Beijing, China; 2 Department of Medical Oncology, Integrated Traditional Chinese and Western Medicine, China-Japan Friendship Hospital, Beijing, China

**Keywords:** clinical outcomes, esophageal cancer, immune checkpoint inhibitors, immune-related adverse events, meta-analysis

## Abstract

**Introduction:**

Immune-related adverse events (irAEs) are distinctive toxicities associated with immune checkpoint inhibitors (ICIs) and have been reported to correlate with clinical outcomes in several cancers. However, whether this association also exists in patients with esophageal cancer (EC) receiving ICIs remains uncertain. This systematic review and meta-analysis aimed to evaluate the association between irAEs and clinical outcomes in this population.

**Methods:**

This systematic review and meta-analysis was conducted and reported in accordance with the Preferred Reporting Items for Systematic Reviews and Meta-Analyses (PRISMA) 2020 statement. PubMed, Embase, Web of Science, and the Cochrane Library were searched from inception to March 2, 2026. Eligible studies evaluated the association between irAEs and progression-free survival (PFS), overall survival (OS), objective response rate (ORR), or disease control rate (DCR). Hazard ratios (HRs) with 95% confidence intervals (CIs) were pooled for PFS and OS, whereas odds ratios (ORs) with 95% CIs were pooled for ORR and DCR using random-effects models. Between-study heterogeneity, 95% prediction intervals (PIs), sensitivity analyses, publication bias, prespecified subgroup analyses, and immortal-time bias were also assessed.

**Results:**

14 studies involving 1, 546 patients were included. The occurrence of irAEs was associated with improved PFS (HR, 0.45; 95% CI, 0.31-0.63), OS (HR, 0.54; 95% CI, 0.46-0.64), ORR (OR, 4.00; 95% CI, 1.58-10.12), and DCR (OR, 3.85; 95% CI, 2.40-6.20). The 95% PIs were 0.16-1.24, 0.44-0.66, 0.19-82.57, and 1.42-10.44 for PFS, OS, ORR, and DCR, respectively, crossing the null value for PFS and ORR. Subgroup estimates were generally similar in direction but remained exploratory. No eligible survival estimates using landmark or time-varying approaches were available for the comparison between patients with and without irAEs.

**Conclusions:**

In patients with EC receiving ICIs, the occurrence of irAEs may be a prognostic marker of favorable outcomes. However, wide PIs for PFS and ORR indicate that these associations may vary across future studies and immortal time bias may overestimate survival associations. This observational evidence derives mainly from East Asian patients with esophageal squamous cell carcinoma and is primarily applicable to these patients. Subgroup findings remain exploratory and require prospective validation.

**Systematic review registration:**

https://www.crd.york.ac.uk/prospero/, identifier CRD420261346294.

## 1 Introduction

Esophageal cancer (EC) is the eleventh most commonly diagnosed cancer and the seventh leading cause of cancer-related death worldwide (1). Esophageal squamous cell carcinoma (ESCC) is the predominant histological subtype in Asia, whereas esophageal adenocarcinoma (EAC) is more common in Western countries (2). EC is typically managed through surgical resection, locoregional therapies or systemic treatments (3, 4). Nevertheless, a large proportion of patients are diagnosed at an advanced stage, resulting in a poor prognosis regardless of the subtype (3).

In recent years, immune checkpoint inhibitors (ICIs) have reshaped the therapeutic landscape of EC, particularly in advanced disease (5). By blocking inhibitory pathways between T lymphocytes and tumor cells or antigen-presenting cells, ICIs aim to release the inhibitory constraints on activated T cells, reactivating their antitumor cytolytic function (6).

The programmed cell death 1/programmed death-ligand 1 (PD-1/PD-L1) axis and cytotoxic T-lymphocyte antigen-4 (CTLA-4) are the principal immune checkpoints targeted in current cancer immunotherapy (7). Because of their mechanism of action, ICIs are associated with a distinctive spectrum of immune-related adverse events (irAEs), which may affect multiple organs and systems, including skin toxicities, myocarditis, hepatitis, colitis, endocrine disorders, inflammatory arthritis, and pneumonitis (8, 9). Growing evidence from studies in solid tumors shows that irAEs may be associated with improved clinical outcomes. In hepatocellular carcinoma, irAEs have been associated with improved treatment efficacy (10). Similarly, irAEs during nivolumab therapy have been associated with improved survival in gastric cancer (11), while studies in gastrointestinal cancers have reported prolonged overall survival (OS) and progression-free survival (PFS) among patients who developed irAEs (12). However, EC is characterized by substantial heterogeneity in histological subtype, geographic distribution, tumor biology, and treatment strategy (13). Therefore, findings from other cancer types may not be directly generalizable to EC.

Accordingly, we performed this systematic review and meta-analysis to investigate whether the occurrence of irAEs is associated with favorable survival outcomes and treatment responses in patients with EC receiving ICIs. By synthesizing the available evidence, we aimed to clarify the association between irAEs and clinical outcomes in EC and to inform clinical interpretation of immunotherapy-associated toxicity.

## 2 Materials and methods

We followed the guidelines outlined in the Preferred Reporting Items for Systematic Review and Meta-Analyses (PRISMA) statement to report this systematic review (14). This protocol was prospectively registered in PROSPERO (ID: CRD420261346294).

### 2.1 Search strategy

Two researchers independently performed a comprehensive systematic literature search in PubMed, Embase, Web of Science, and the Cochrane Library to identify studies evaluating the association between the occurrence of irAEs and clinical outcomes in patients with EC receiving ICIs. The search covered all records from database inception to March 2, 2026. The main keywords used for search included immune-related adverse effects, irAEs, ICIs, PD-1, PD-L1, CTLA-4, effectiveness, prognosis and EC. The complete search strategy is provided in Text S1. Any disagreements were resolved by discussion and consensus. The completed PRISMA 2020 checklist is provided in **Supplementary Table S1**.

### 2.2 Inclusion and exclusion criteria

Studies were considered eligible if they: (1) included patients with EC; (2) received treatment with ICIs, either as monotherapy or in combination with other anticancer therapies; (3) reported the association between irAEs and clinical outcomes, including at least one of the following results: hazard ratios (HRs) and 95% confidence intervals (CIs) of PFS or OS in patients with irAEs versus patients without irAEs, or objective response rate (ORR) or disease control rate (DCR) for patients with and without irAEs.

Studies were excluded if they: (1) reported adverse events not related to immune disorder; (2) had duplicate data or overlapping study populations; (3) reported insufficient needed data or information available; (4) published as case reports, editorials, reviews or animal experiments.

Full-text articles and conference abstracts were both considered eligible when sufficient data were available for extraction.

### 2.3 Data collection and quality assessment

Two researchers independently extracted data from eligible studies using a predefined electronic spreadsheet, and discrepancies were resolved by consensus. The following information was collected: first author, publication year, country, study design, sample size, pathological type, line of therapy, details of combination therapy, ICIs regimen, criteria for grading irAEs, statistical model, follow-up duration, irAEs type and grade. Outcome data included HRs with 95% CIs for PFS and OS comparing patients with and without irAEs, and ORR or DCR data for both groups. When both adjusted and unadjusted HRs were reported, adjusted estimates were preferentially extracted. When both a conference abstract and a subsequent full-text publication reported overlapping data, only the report with the most complete information was included. When studies enrolled mixed upper gastrointestinal cancer populations, only data comparing patients with EC who developed irAEs with those who did not were extracted.

ORR was defined as the proportion of patients who achieved a complete response (CR) or partial response (PR), whereas DCR was defined as the proportion who achieved CR, PR, or stable disease (SD), according to the Response Evaluation Criteria in Solid Tumors (RECIST).

Methodological quality was assessed using the Newcastle-Ottawa Scale (NOS) (15). Two researchers independently evaluated each study for cohort selection, comparability, and outcome assessment, with disagreements resolved by consultation with a third author. NOS scores ranged from 0 to 9, with higher scores indicating better methodological quality. Studies that provided data allowing estimation of the association between irAEs and PFS or OS among patients receiving ICIs, either directly or indirectly, were considered eligible for inclusion.

### 2.4 Data analysis

The meta-analysis was performed using the meta package (version 8.2-1) in R (version 4.5.2), following established methodological guidance (16), with the variance between studies (τ²) estimated using restricted maximum likelihood (REML). We compared survival and treatment response between patients with and without irAEs. For survival outcomes, HR and 95% CI for PFS and OS were used as the effect measures. If a study reported HR and p values but not 95% CI, the method described by Altman was used to calculate the 95% CI (17). If an HR for patients without irAEs versus patients with irAEs, rather than the opposite comparison, was reported, an HR for patients with irAEs versus patients without irAEs was calculated by taking the reciprocal of the original HR and corresponding CI (18). If HRs were not reported but the Kaplan-Meier curve was available, HR for PFS or OS were estimated using the method proposed by Liu et al. (19). For treatment response outcomes, odds ratios (OR) and 95% CI for ORR and DCR were calculated from the numbers of patients who achieved versus did not achieve objective response or disease control among patients with and without irAEs. When the HR was less than 1 and the upper bound of its 95% CI was also below 1, it indicated improved survival in patients with irAEs, whereas the OR greater than 1 and the lower bound of its 95% CI was also greater than 1, it indicated superior treatment response.

Heterogeneity was assessed using the I² statistic, with I² values of <25%, 25%-75%, and >75% representing low, moderate, and high heterogeneity, respectively (20). In addition, we report the 95% prediction intervals (PI). The 95% PI provides a prediction range for a single future study; 95% PI including the null value of 1 indicates uncertainty regarding whether the association would be consistently observed in future studies (21). Random-effects models were used for all pooled analyses to account for study variability (22). Publication bias was assessed visually using funnel plots and statistically using Begg’s and Egger’s tests. Where appropriate, the trim-and-fill method was applied to overcome potential minimal publication bias. Sensitivity analyses were performed by sequentially omitting one study at a time to assess the robustness of the pooled estimates. All statistical tests were two-sided, and p < 0.05 was considered statistically significant.

For the overall pooled analyses, only HRs for global irAEs and estimates comparing patients with all-grade irAEs versus patients without irAEs were included; organ-specific HRs and grade-specific estimates were excluded. Predefined subgroup analyses were conducted to explore whether the association between irAEs and clinical outcomes varied across different study settings and to identify potential sources of heterogeneity. The following subgroup variables were considered: statistical model, combination therapy, ICIs regimen, pathological type, sample size, line of therapy, and country. For each outcome, subgroup analyses in which any category included only one study were presented in full in the **Supplementary Material**, whereas the remaining subgroup analyses were presented in the main text. Estimates from a single study were displayed as gray markers with dashed lines for their 95% CIs and interpreted descriptively. No formal between-subgroup comparison was made.

We also assessed potential immortal-time bias in studies reporting PFS or OS because irAEs are post-treatment, time-dependent events. For each study, we examined the time zero for survival analysis, the reported timing or ascertainment window of irAEs, the survival analysis method, whether landmark or time-varying approaches were used to address this bias. For EC-specific comparisons between patients with and without irAEs, immortal-time bias was considered adequately addressed only when a landmark approach defined irAEs status and the at-risk population at the landmark, with survival measured from that time point, or when a time-varying Cox regression model treated the occurrence of irAEs as a time-dependent covariate. We then determined whether sufficient eligible estimates were available for a sensitivity analysis restricted to studies meeting these criteria; outcomes with fewer than two eligible estimates were summarized descriptively.

## 3 Results

### 3.1 Literature search and screening

As shown in **Figure 1**, the literature search yielded 2, 182 records. After screening titles and abstracts, 28 articles were considered potentially eligible and underwent full-text review. Ultimately, 14 studies comprising 1, 546 patients were included in the final analysis (23–36).

**Figure 1:**
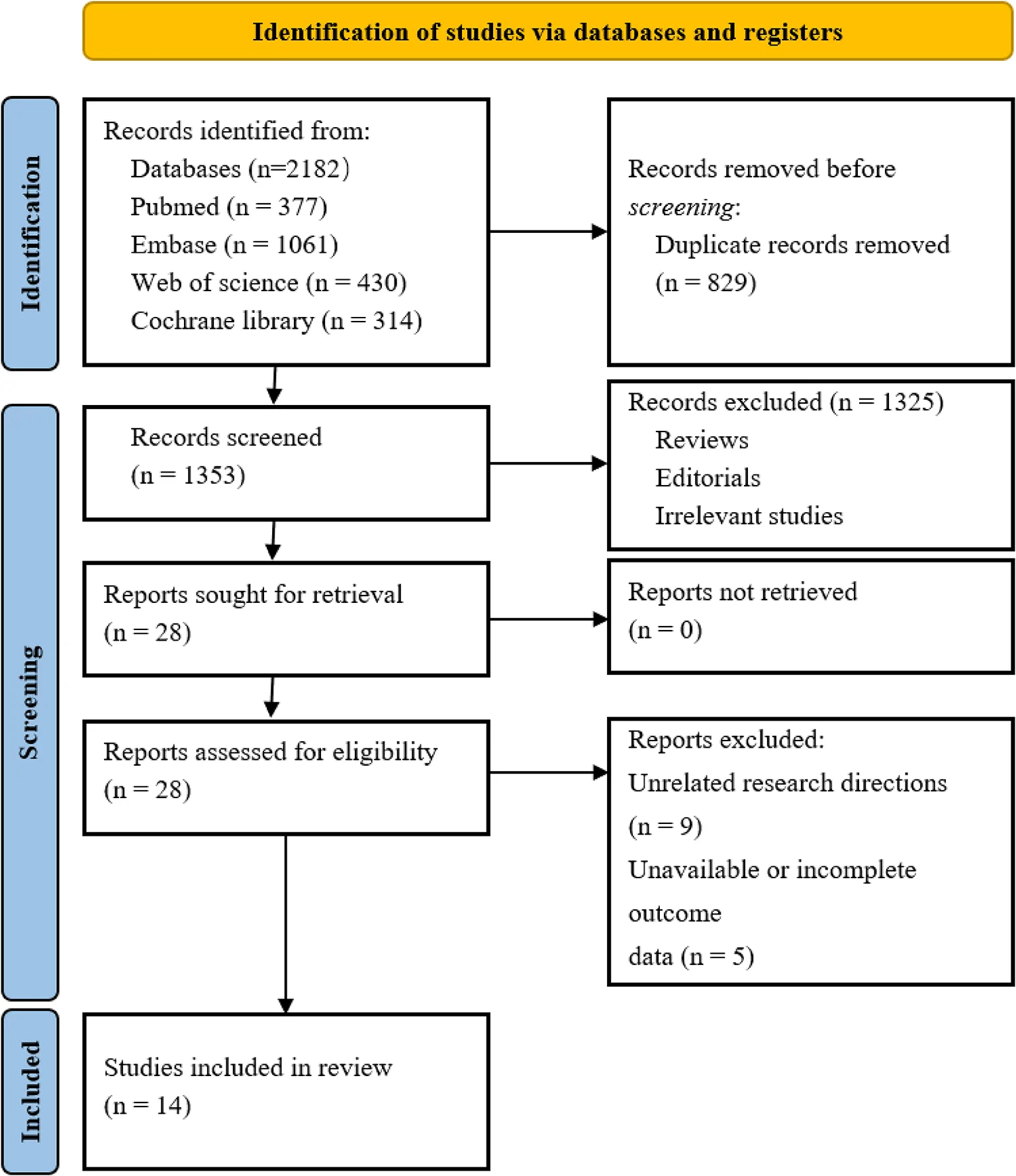
Flow diagram of the primary studies selection process.

### 3.2 Characteristics of the included studies

The characteristics of the included studies were summarized in **Table 1**, **Supplementary Tables S2–S4**. Of the 14 included studies, 13 were retrospective (23–33, 35, 36) and 1 was prospective (34); with sample sizes ranging from 19 to 397. A total of 10 studies included PFS data (23–25, 27–29, 31–34), 10 provided OS data (23–25, 27–29, 32–34, 36), and 6 provided ORR (24, 26, 30, 32, 35, 36) and DCR data (24, 26, 30, 32, 35, 36), respectively. By geographic region, 7 studies were conducted in China (24, 29, 32–36), 6 studies in Japan (23, 26–28, 30, 31), and 1 study in the United States (25). In terms of histological type, 9 studies focused on ESCC (23, 24, 26, 28–30, 34–36), while 5 studies included a mix of histological types (25, 27, 31–33). Regarding ICIs regimens, most eligible studies evaluated PD-1 inhibitors (23, 24, 26–30, 32–36), only two studies explored PD-1 combined with CTLA-4 therapy (25, 31). In terms of concomitant therapy, 6 studies included no additional anticancer therapy (23, 26–28, 31, 36), 2 included anticancer therapy in all patients (34, 35), 5 studies included anticancer therapy in a subset of patients (24, 25, 29, 32, 33), and 1 study did not provide this information (30). Regarding line of therapy, 9 studies included patients treated across different lines of therapy (24–29, 32, 33, 36), 3 studies involved first-line therapy (31, 34, 35), and 2 studies did not report the therapy line (23, 30). Among the statistical analyses of PFS outcomes, 3 studies involved multivariable analyses (24, 31–32) and 7 studies involved univariate analysis (23, 25, 27–29, 33, 34); among the statistical analyses of OS outcomes, 4 studies involved multivariable analyses (24, 32, 33, 36) and 6 involved univariate regression analysis; respectively (23, 25, 27–29, 34). 9 studies (24, 26, 30–36) provided types of irAEs of all-grade and 7 studies (24, 26, 32–36) provided the incidence rates of types of irAEs of all-grade. Skin-related and endocrine-related irAEs were the most frequently reported all-grade events. 7 studies (24, 26, 28, 32–34, 36) provided the incidence rates and types of high-grade, among which pneumonia and elevated transaminases/hepatitis were the most common.

**Table 1:** Main characteristics of the included primary studies.

Study	Country	Median follow-up time (months)	Sample size	Pathological type	Line of therapy	ICIs regimen	Combination therapy	PFS statistical model	OS statistical model
Booka 2021 (23)	Japan	NA	21	ESCC	NA	PD-1	No	Unadjusted	Unadjusted
Cao 2023 (24)	China	NA	78	ESCC	Mixed	PD-1	Mixed	Adjusted	Adjusted
Hamai 2024 (26)	Japan	NA	82	ESCC	Mixed	PD-1	No	NA	NA
Hara 2022 (27)	Japan	NA	61	Mixed	Mixed	PD-1	No	Unadjusted	Unadjusted
Hirasawa 2024 (28)	Japan	8.3	86	ESCC	Mixed	PD-1	No	Unadjusted	Unadjusted
Ma 2025 (29)	China	NA	42	ESCC	Mixed	PD-1	Mixed	Unadjusted	Unadjusted
Nakada 2021 (30)	Japan	NA	34	ESCC	NA	PD-1	NA	NA	NA
Qiu 2025 (33)	China	18.2	397	Mixed	Mixed	PD-1	Mixed	Unadjusted	Adjusted
Wang 2024 (34)	China	44.3	42	ESCC	First-line	PD-1	Yes	Unadjusted	Unadjusted
Zhao 2023 (36)	China	26.0	136	ESCC	Mixed	PD-1	No	NA	Adjusted
Qin 2022 (32)	China	NA	295	Mixed	Mixed	PD-1	Mixed	Adjusted	Adjusted
Natsuki 2025 (31)	Japan	10.0	19	Mixed	First-line	PD-1+CTLA-4	No	Unadjusted	NA
Elghawy 2026 (25)	United States	NA	40	Mixed	Mixed	PD-1+CTLA-4	Mixed	Unadjusted	Unadjusted
Zhang 2026 (35)	China	NA	213	ESCC	First-line	PD-1	Yes	NA	NA

NA, not reported; ESCC, esophageal squamous cell carcinoma; ICIs, immune checkpoint inhibitors; PD-1, programmed cell death 1; CTLA-4, cytotoxic T-lymphocyte antigen-4. “Mixed” indicates that multiple categories were included within a study (e.g., histological subtypes of EC, line of therapy, or combination therapy) without stratified reporting. Combination therapy refers to ICIs administered in combination with other anticancer therapy (e.g., chemotherapy, radiotherapy, or targeted therapy), excluding dual immune checkpoint blockade (e.g., PD-1 plus CTLA-4). For combination therapy, “Mixed” indicates that only a subset of patients received additional anticancer therapy; this proportion was 95.2% (40/42) in Ma et al. and was not reported in the other studies within this category. “Statistical model classification” refers to the adjustment status of the HRs for PFS and OS included in this meta-analysis. Adjusted indicates the HR derived from a multivariate model, while unadjusted indicates the HRs estimated without adjusting for other covariates.

The quality of each study was assessed using the NOS criteria, with scores ranging from 7 to 9. Detailed scoring results are provided in **Supplementary Table S2**, **Supplementary Figure S1**. All included studies met the predefined quality threshold and were therefore retained in the meta-analysis.

Among the included studies, no published PFS or OS estimates for the EC-specific comparison of interest were derived using landmark or time-varying approaches (**Supplementary Table S5**). Accordingly, a restricted sensitivity meta-analysis was not feasible.

### 3.3 Association between irAEs and clinical outcomes

#### 3.3.1 OS and PFS

Pooled analyses of primary studies on PFS and OS showed that the occurrence of irAEs was associated with prolonged PFS (HR, 0.45; 95% CI, 0.31-0.63) and OS (HR, 0.54; 95% CI, 0.46-0.64). The 95% PI for PFS included the null value (0.16-1.24), suggesting that this association may not be consistently replicated in future studies, whereas the 95% PI for OS suggested greater consistency in the direction of the association (0.44-0.66) (**Figure 2**).

**Figure 2:**
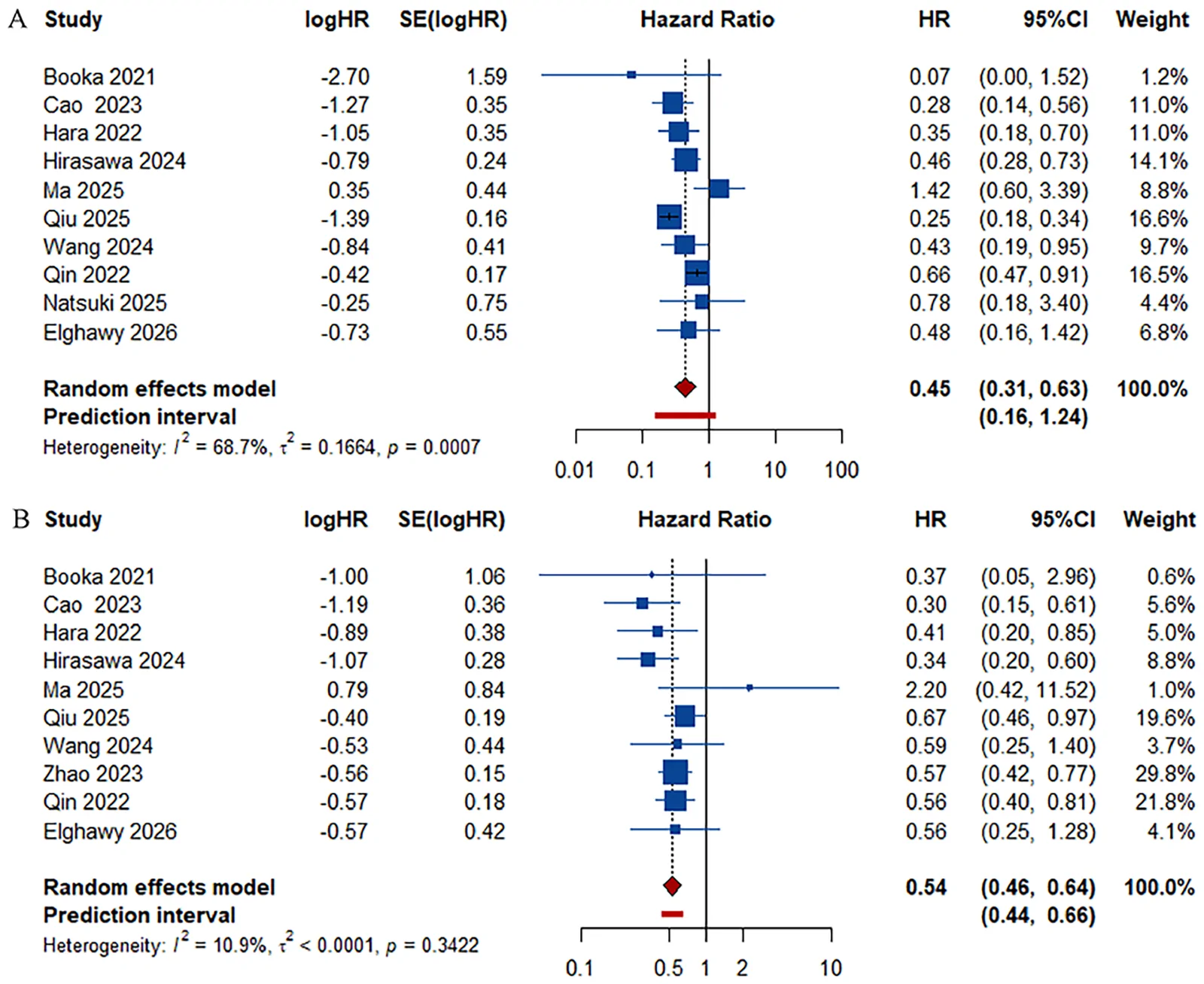
Forest plot of the association between irAEs and survival outcome. **(A)** PFS; **(B)** OS. irAEs, immune-related adverse events; OS, overall survival; PFS, progression-free survival.

Predefined subgroup analyses were conducted according to study characteristics and patient-related factors. For adjusted and unadjusted estimates, the pooled HRs were 0.50 (95% CI, 0.27-0.95) and 0.42 (95% CI, 0.27-0.66) for PFS and 0.46 (95% CI, 0.33-0.65) and 0.57 (95% CI, 0.47-0.68) for OS, respectively (**Figure 3**). Although all subgroup point estimates were below 1, the 95% CIs for PFS crossed 1 in the PD-1 plus CTLA-4 subgroup and in studies enrolling more than 200 patients (**Figure 3**). Additional exploratory analyses are presented in **Supplementary Figure S2**. The estimates for the United States were derived from a single study and do not support comparisons between regions. More broadly, all subgroup analyses were exploratory and should be interpreted with caution.

**Figure 3:**
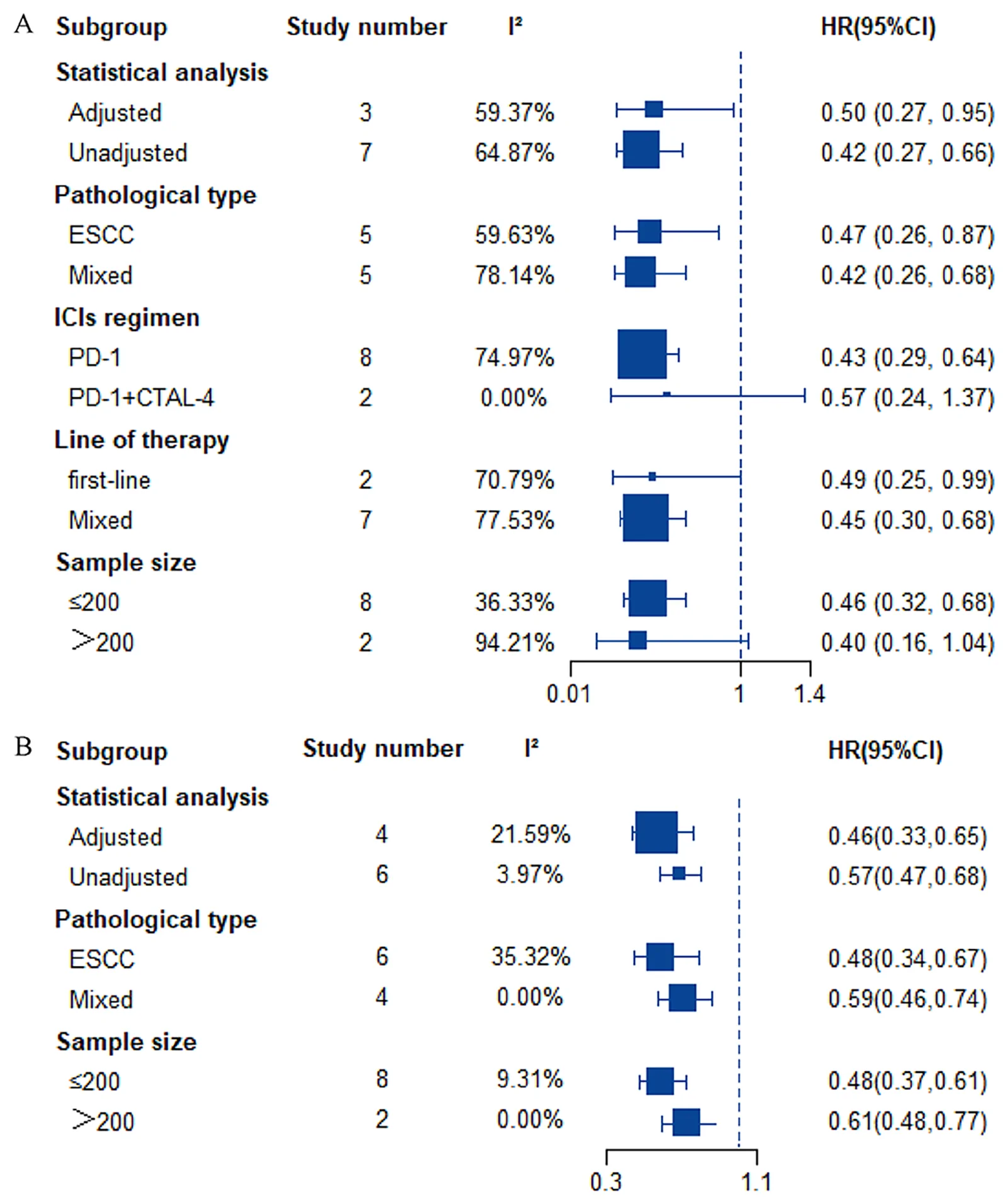
Subgroup analyses of the association between irAEs and survival outcome. **(A)** PFS; **(B)** OS. irAEs, immune-related adverse events; OS, overall survival; PFS, progression-free survival. Only analyses in which every category included at least two studies are shown.

#### 3.3.2 ORR and DCR

Pooled analysis showed that, among patients with EC receiving ICIs, patients with irAEs had higher ORR (OR, 4.00; 95% CI, 1.58-10.12) and DCR (OR, 3.85; 95% CI, 2.40-6.20) than patients without irAEs. The 95% PI included the null value for ORR (95% PI, 0.19-82.57), but not for DCR (95% PI, 1.42-10.44), suggesting greater uncertainty for ORR than for DCR in future studies (**Figure 4**).

**Figure 4:**
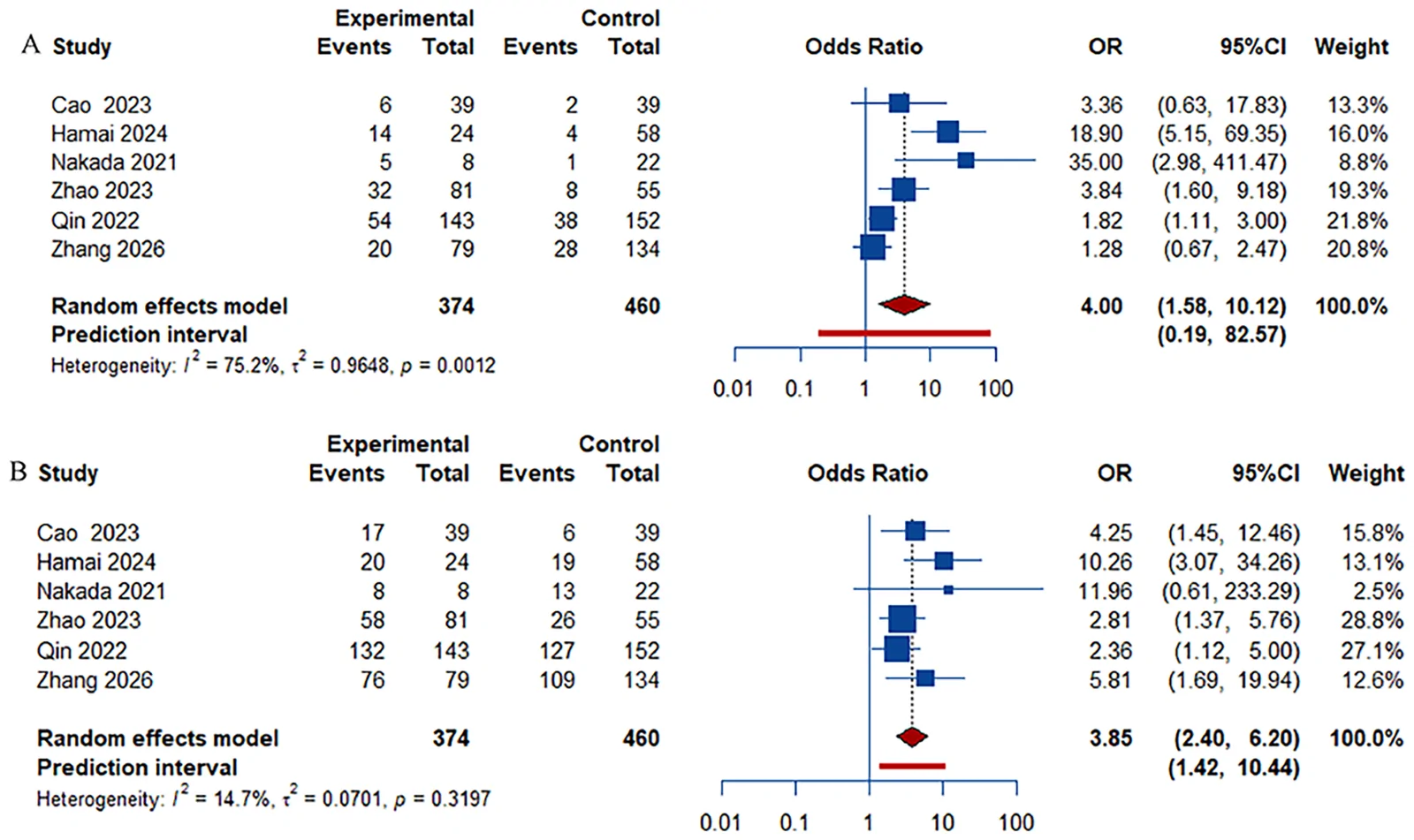
Forest plot of the association between irAEs and treatment response. **(A)** ORR; **(B)** DCR. irAEs, immune-related adverse events; ORR, objective response rate; DCR, disease control rate.

These associations were further examined in subgroup analyses according to study characteristics. Pooled ORs for ORR and DCR exceeded 1 across the country and sample size subgroups, although their magnitude and precision varied (**Figure 5**). Additional subgroup analyses are presented in **Supplementary Figure S3**. Given the limited number of studies, including categories represented by a single study, these findings remain exploratory and do not support firm conclusions about differences or consistency between subgroups.

**Figure 5:**
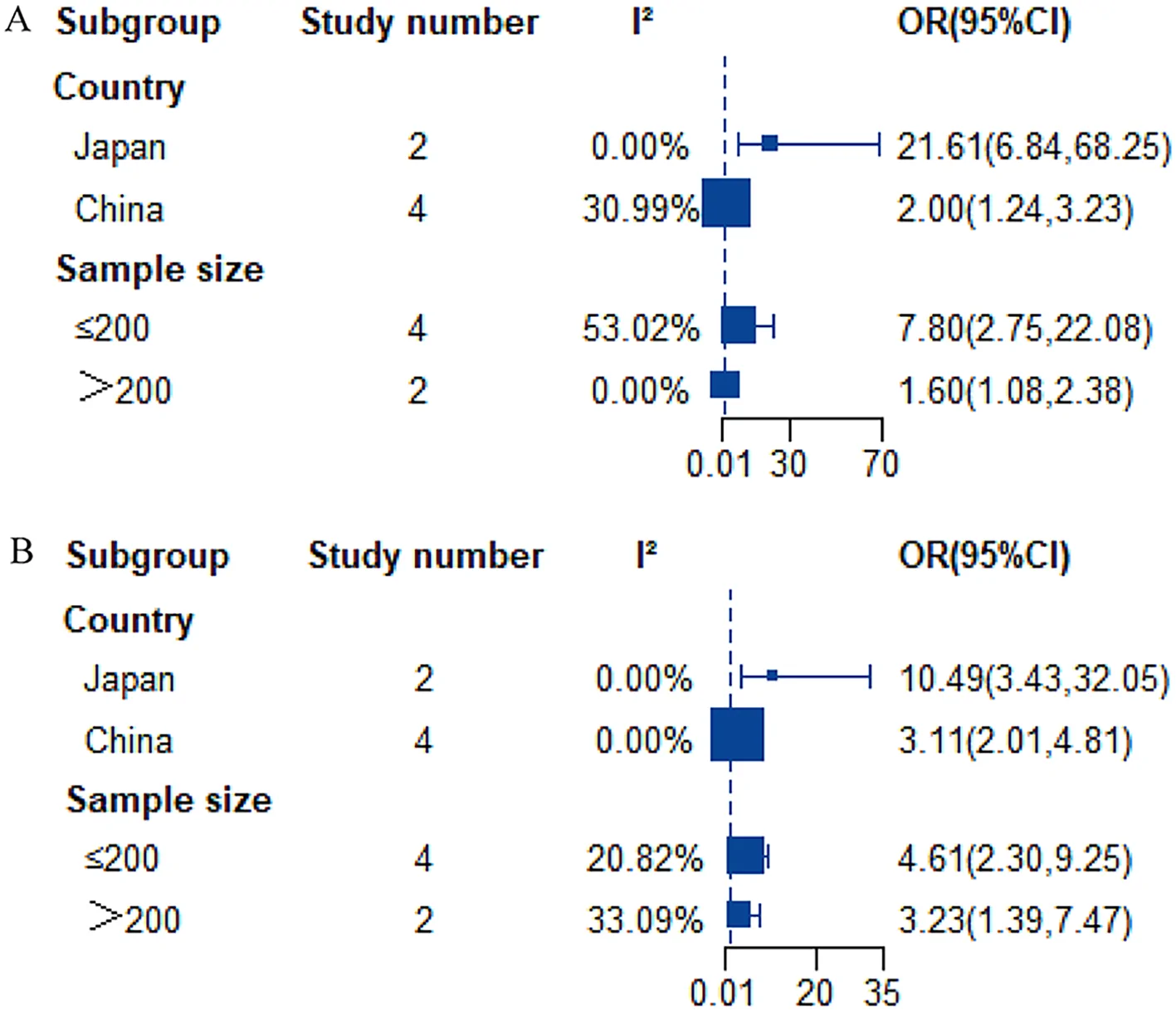
Subgroup analyses of the association between irAEs and treatment response. **(A)** ORR; **(B)** DCR. irAEs, immune-related adverse events; ORR, objective response rate; DCR, disease control rate. Only analyses in which every category included at least two studies are shown.

### 3.4 Descriptive analysis of non-pooled evidence from included studies

Due to substantial heterogeneity in reporting formats and outcome definitions, several studies were not eligible for quantitative synthesis and were therefore included in the descriptive analysis. Although not included in the meta-analysis, these studies provided supplementary evidence regarding the potential association between irAEs and clinical outcomes.

When stratified by irAEs type, endocrine irAEs were more consistently associated with favorable clinical outcomes, including higher DCR and prolonged PFS and OS, and were identified as independent protective factors in multivariable analyses (24, 35). In contrast, findings for cutaneous irAEs were inconsistent across studies (24, 31, 35), and the associations for other organ-specific or systemic irAEs were less consistent (31, 35). Regarding severity, low-grade irAEs were generally associated with favorable clinical outcomes and were identified as independent protective factors in several studies (26, 35), whereas high-grade irAEs did not show a consistent survival benefit (24). With respect to timing, some studies reported an association between early-onset irAEs and improved clinical outcomes (26, 32), whereas others found no significant difference between early- and late-onset irAEs (24, 31). In terms of the number of irAEs, some studies reported greater survival benefit in patients with multiple irAEs than in those with a single or no irAEs (31, 32), whereas others found no significant difference between single and multiple irAEs (34, 35). Limited evidence also suggested that the association between irAEs and clinical outcomes might differ according to PD-L1 expression status (36).

In summary, the descriptive evidence showed that the association between irAEs and clinical outcomes varied according to irAE type, severity, timing of onset, number of irAEs, and PD-L1 expression status, although the overall trend favored better outcomes in patients with irAEs.

### 3.5 Sensitivity analysis and publication bias

In sensitivity analysis, the pooled results for OS, PFS, ORR and DCR remained statistically significant regardless of which study was excluded, suggesting that no individual study materially influenced the pooled estimates (**Supplementary Figure S4**).

The publication bias was assessed using funnel plots and statistically by Begg’s and Egger’s tests. Both tests yielded nonsignificant results for PFS, OS, ORR, and DCR (**Supplementary Figure S5**; **Table 2**). In addition, we performed trim-and-fill analyses. The ORR association was attenuated and no longer significant (OR, 1.75; 95% CI, 0.55–5.55), whereas those for PFS, OS, and DCR remained significant (**Supplementary Table S6**). Publication bias cannot be excluded given the limited power of Begg’s and Egger’s tests when few studies are available.

**Table 2:** Results of publication bias.

Item	Begg’s tests	Egger’s tests
PFS	0.655	0.793
OS	0.929	0.743
ORR	0.186	0.062
DCR	0.091	0.070

## 4 Discussion

In this meta-analysis, we comprehensively evaluated the association between irAEs and clinical outcomes in patients with EC receiving ICIs. Our pooled results showed that the occurrence of irAEs may serve as a prognostic marker associated with improved clinical outcomes, including prolonged PFS and OS, as well as higher ORR and DCR. The pooled estimates were not materially altered in sensitivity analyses, while similar effect directions were observed across most evaluable subgroups. However, wide 95% PIs, particularly for PFS and ORR, indicate uncertainty across future comparable studies. The observational evidence remains susceptible to immortal-time bias, which may exaggerate survival associations. Because 13 of the 14 studies were conducted in East Asia and mainly involved patients with ESCC, the generalizability of our findings to Western patients with EAC is substantially limited.

Our findings are broadly consistent with previous meta-analyses in other tumors (10, 11, 37, 38), which reported associations between irAEs and improved survival or treatment response. However, evidence specific to EC remains relatively limited and heterogeneous, with available studies differing in statistical model, combination therapy, ICIs regimen, pathological type, line of therapy, and country. This meta-analysis provides disease-specific evidence for interpreting the clinical relevance of irAEs in patients with EC receiving ICIs. Beyond the overall association between the occurrence of irAEs and clinical outcomes, identifying which irAEs phenotypes are associated with greater clinical benefit may be more clinically meaningful.

Previous meta-analyses in several cancers, including lung and liver cancers, have reported that endocrine and cutaneous irAEs are often associated with better clinical outcomes among patients receiving ICIs (10, 37, 39–41). In our non-pooled evidence synthesis, endocrine irAEs showed a relatively consistent association with improved survival or treatment response, indicating that they may serve as a potentially informative organ-specific irAEs phenotype in patients with EC receiving ICIs. In contrast, findings for cutaneous irAEs were not fully consistent, possibly because of differences in the definitions of cutaneous irAEs across studies and variations in the ICIs agents used. The severity and number of irAEs should also be considered when interpreting their association with clinical outcomes. Low-grade irAEs are generally associated with favorable clinical outcomes, whereas high-grade irAEs have not shown a consistent survival or treatment benefit, which is broadly consistent with previous studies (10, 37–40). Therefore, more severe irAEs do not necessarily indicate greater clinical benefit, and their clinical significance should be interpreted in the context of toxicity severity.

The timing of irAEs onset must also be considered. Previous studies have reported inconsistent findings regarding the association between irAEs onset timing and clinical outcomes. Some studies have suggested that early- or late-onset irAEs may be associated with improved treatment response and survival outcomes (42–44), whereas others have not observed significant differences between early- and late-onset irAEs (45). In the included studies, early-onset irAEs appeared to be associated with higher tumor response rates and longer survival. However, irAEs are treatment-emergent events that occur after the initiation of ICIs therapy rather than baseline factors present before treatment. Therefore, direct comparisons of outcomes between patients who develop irAEs and those who do not, particularly when the timing of irAEs onset is not adequately accounted for, may be susceptible to time-dependent bias. Accordingly, the association between irAEs onset timing and favorable clinical outcomes should be interpreted with caution.

In addition, some studies have reported that patients with multiple irAEs may exhibit better antitumor responses than those with a single irAE or no irAEs (38, 46, 47). However, the evidence regarding multiple irAEs in the included studies remains limited, and these findings should therefore be considered exploratory. Moreover, limited evidence from a single included study suggested that the association between irAEs and OS may differ according to PD-L1 expression status; however, this finding remains preliminary and requires further validation. Overall, the occurrence of irAEs may serve as an observable marker of immune activation in patients receiving ICIs. The clinical outcome associated with irAEs may depend not only on whether they occur, but also on the affected organ system, severity, timing of irAEs assessment, number of events, and the underlying tumor immune microenvironment. Therefore, irAEs should not be interpreted as a therapeutic target, and severe toxicities should not be tolerated in pursuit of potential clinical benefits.

The precise mechanisms underlying the association between irAEs and clinical outcomes remain incompletely understood. One of the most commonly posited hypotheses is the antigen mimicry theory, which postulates that tumor antigens and normal tissue antigens may share common antigens or similar epitopes (48). As a result, T cells activated by ICIs may not only target tumor cells but also recognize normal tissues expressing shared or similar antigens, leading to potentially cross-reactive immune responses. This mechanism has been most extensively described in melanoma. Because melanoma cells share differentiation antigens with normal melanocytes, the development of vitiligo-like depigmentation following treatment is often regarded as a clinical manifestation of enhanced anti-melanocyte immunity and has been associated with prolonged survival (49–51). Similar observations have also been reported in non-small cell lung cancer (52). Another possible explanation is that irAEs may reflect robust and sustained immune activation induced by ICIs (53). By restoring T-cell-mediated antitumor activity, ICIs may simultaneously disrupt immune tolerance to normal tissues, thereby leading to irAEs. In this context, the occurrence of irAEs may serve as a clinical indicator of intensified antitumor immunity, which may in turn contribute to improved clinical outcomes (40, 48). However, this interpretation remains hypothetical and does not establish a causal relationship. Humoral immune responses may also play a role in the development of irAEs and their association with clinical outcomes (54). Previous studies have shown that patients with autoantibody positivity at baseline or during treatment may have a higher risk of developing irAEs and a more favorable clinical outcomes (55, 56), suggesting that susceptibility to autoimmunity and enhanced antitumor immune activation may share overlapping biological mechanisms. However, this hypothesis still requires further validation. In addition, the relationship between irAEs and clinical outcomes is potentially linked to various mechanisms, including tumor microenvironment, systemic inflammation and cytokine networks, gut microbiota dysbiosis, and the immune-host genetic background (57–61).

Subgroup analyses generally showed similar effect directions across most evaluable settings. Nevertheless, these findings were exploratory and should be interpreted cautiously. Several estimates were based on few studies, with some strata represented by a single study, limiting reliable comparisons across subgroups. In addition, wide 95% CIs in some subgroups indicated limited precision, particularly for analyses by combination therapy and country. Without formal interaction tests, these findings neither demonstrate consistency across subgroups nor exclude clinically meaningful differences that may have remained undetected because of limited statistical power.

In the pooled analysis, heterogeneity was observed for both PFS (I² = 68.660%) and ORR (I² = 75.194%), which may partly explain why the 95% PIs for these outcomes crossed the null value despite statistically significant pooled estimates (62). This variability may be attributable to multiple factors, including differences in histological subtype (e.g., ESCC vs. EAC), ICIs regimen, use of combination therapy, baseline treatment characteristics, tumor stage, study design or statistical methods, the timing and frequency of irAEs assessment, irAEs management including corticosteroid use, baseline PD-L1 expression. Although subgroup analyses showed generally similar trends, they did not fully account for the observed heterogeneity. Residual heterogeneity may still reflect the influence of unmeasured confounding factors and inconsistencies in reporting across studies. The wide PIs, particularly for ORR and PFS, indicate that the magnitude and direction of these associations may vary across future comparable studies.

Several limitations of this study should be acknowledged. First, most included studies were retrospective in nature, which may have increased susceptibility to bias, particularly selection bias and recall bias. Second, when HRs were not directly reported, they were estimated from Kaplan-Meier curves, which may have introduced minor statistical inaccuracies. Third, evidence for certain subgroups, such as patients receiving anti-CTLA-4 therapy or combination therapy, was limited, thereby restricting the robustness of these subgroup analyses. In addition, some included studies were available only as conference abstracts, which may provide limited methodological details and incomplete outcome reporting. Besides, given that 13 of the 14 included studies were conducted in East Asia and primarily enrolled patients with ESCC, the generalizability of our findings to Western patients with EAC is severely limited. Finally, time-dependent bias remains an important methodological concern in this field (63, 64).

Because irAEs occur after treatment initiation, patients who develop irAEs must survive until their onset. Treating irAE status as a baseline characteristic assigns the period before irAE onset to the exposed group, potentially exaggerating survival benefit. This bias may have shifted the HRs for PFS and OS downward, making the observed associations appear stronger than they truly are. Accordingly, these findings should be interpreted as exploratory prognostic associations and do not establish causality or independent predictive value. Future studies should preferentially incorporate landmark analyses or Cox models with time-dependent covariates to account for irAEs onset and reduce the risk of immortal-time bias.

## 5 Conclusions

In this systematic review and meta-analysis, irAEs were associated with longer survival and higher treatment response in patients with EC receiving ICIs, suggesting that they may serve as a prognostic marker of favorable outcomes. However, wide PIs for PFS and ORR indicate uncertainty in future studies, and the observational evidence remains susceptible to immortal-time bias. As these findings were derived predominantly from East Asian patients with ESCC, they are primarily applicable to these patients. Further prospective studies in diverse populations using landmark or time-varying approaches are warranted to clarify the prognostic significance of irAEs.

## Data Availability

The original contributions presented in the study are included in the article/**Supplementary Material**. Further inquiries can be directed to the corresponding author.

## References

[1] Bray F Laversanne M Sung H Ferlay J Siegel RL Soerjomataram I . Global cancer statistics 2022: GLOBOCAN estimates of incidence and mortality worldwide for 36 cancers in 185 countries. CA Cancer J Clin. (2024) 74:229–63. doi: 10.3322/caac.21834 38572751

[2] Jiang W Zhang B Xu J Xue L Wang L . Current status and perspectives of esophageal cancer: a comprehensive review. Cancer Commun (Lond). (2025) 45:281–331. doi: 10.1002/cac2.12645 39723635 PMC11947622

[3] Deboever N Jones CM Yamashita K Ajani JA Hofstetter WL . Advances in diagnosis and management of cancer of the esophagus. BMJ. (2024) 385:e074962. doi: 10.1136/bmj-2023-074962 38830686

[4] Jamal A Aldreiwish AD Banawas SS Alqurashi YE Kamal MA Ahmad F . The paths toward immunotherapy of esophageal cancer: an overview of clinical trials. Int Immunopharmacol. (2025) 151:114261. doi: 10.1016/j.intimp.2025.114261 40015204

[5] Chen Z Song Z Den S Zhang W Han M Lan T . Application of immune checkpoint inhibitors in cancer. MedComm (2020). (2025) 6:e70176. doi: 10.1002/mco2.70176 40787068 PMC12335673

[6] Sun Q Hong Z Zhang C Wang L Han Z Ma D . Immune checkpoint therapy for solid tumours: clinical dilemmas and future trends. Signal Transduct Target Ther. (2023) 8:320. doi: 10.1038/s41392-023-01522-4 37635168 PMC10460796

[7] Su X Li J Xu X Ye Y Wang C Pang G . Strategies to enhance the therapeutic efficacy of anti-PD-1 antibody, anti-PD-L1 antibody and anti-CTLA-4 antibody in cancer therapy. J Transl Med. (2024) 22:751. doi: 10.1186/s12967-024-05552-6 39123227 PMC11316358

[8] Kato K Doki Y Chau I Xu J Wyrwicz L Motoyama S . Nivolumab plus chemotherapy or ipilimumab versus chemotherapy in patients with advanced esophageal squamous cell carcinoma (CheckMate 648): 29-month follow-up from a randomized, open-label, phase III trial. Cancer Med. (2024) 13:e7235. doi: 10.1002/cam4.7235 38716626 PMC11077338

[9] Qin J Xue L Hao A Guo X Jiang T Ni Y . Neoadjuvant chemotherapy with or without camrelizumab in resectable esophageal squamous cell carcinoma: the randomized phase 3 ESCORT-NEO/NCCES01 trial. Nat Med. (2024) 30:2549–57. doi: 10.1038/s41591-024-03064-w 38956195 PMC11405280

[10] Liu S Li Z Lin J Ke L Hua Y . Meta-analysis: immune-related adverse events are associated with improved effectiveness of immune checkpoint inhibitors in hepatocellular carcinoma. Aliment Pharmacol Ther. (2026) 63:188–202. doi: 10.1111/apt.70427 41116751

[11] Zhang YJ Tian QY Wang CE . Association between immune-related adverse events and the prognosis of patients with gastric cancer treated with nivolumab: a meta-analysis. Front Oncol. (2024) 14:1408755. doi: 10.3389/fonc.2024.1408755 39301552 PMC11410568

[12] Yan W Qin L Han Y Jia X Wu J . Impact of immune-related adverse events on survival in patients with gastrointestinal cancer treated with immune checkpoint inhibitors: a meta-analysis. Clin Transl Gastroenterol. (2025) 16:e00875. doi: 10.14309/ctg.0000000000000875 40526107 PMC12456484

[13] Zhang S Shen Y Zhu L Lai Y Zhu L Xiao X . Esophageal cancer: from pathogenesis to precision therapies. Signal Transduct Target Ther. (2026) 11:134. doi: 10.1038/s41392-026-02614-7 41986343 PMC13083882

[14] Page MJ McKenzie JE Bossuyt PM Boutron I Hoffmann TC Mulrow CD . The PRISMA 2020 statement: an updated guideline for reporting systematic reviews. Syst Rev. (2021) 10:89. doi: 10.1186/s13643-021-01626-4 33781348 PMC8008539

[15] Stang A . Critical evaluation of the Newcastle-Ottawa scale for the assessment of the quality of nonrandomized studies in meta-analyses. Eur J Epidemiol. (2010) 25:603–5. doi: 10.1007/s10654-010-9491-z 20652370

[16] Balduzzi S Rucker G Schwarzer G . How to perform a meta-analysis with R: a practical tutorial. Evid Based Ment Health. (2019) 22:153–60. doi: 10.1136/ebmental-2019-300117 31563865 PMC10231495

[17] Altman DG Bland JM . How to obtain the confidence interval from a P value. BMJ. (2011) 343:d2090. doi: 10.1136/bmj.d2090 21824904

[18] Tierney JF Stewart LA Ghersi D Burdett S Sydes MR . Practical methods for incorporating summary time-to-event data into meta-analysis. Trials. (2007) 8:16. doi: 10.1186/1745-6215-8-16 17555582 PMC1920534

[19] Liu N Zhou Y Lee JJ . IPDfromKM: reconstruct individual patient data from published Kaplan-Meier survival curves. BMC Med Res Methodol. (2021) 21:111. doi: 10.1186/s12874-021-01308-8 34074267 PMC8168323

[20] Higgins JP Thompson SG . Quantifying heterogeneity in a meta-analysis. Stat Med. (2002) 21:1539–58. doi: 10.1002/sim.1186 12111919

[21] IntHout J Ioannidis JP Rovers MM Goeman JJ . Plea for routinely presenting prediction intervals in meta-analysis. BMJ Open. (2016) 6:e010247. doi: 10.1136/bmjopen-2015-010247 27406637 PMC4947751

[22] Higgins JPT Thomas J Chandler J Cumpston M Li T Page MJ . Cochrane Handbook for Systematic Reviews of Interventions Version 6.2 (Updated February 2021) (2021). Cochrane. Available online at: www.cochrane.org/authors/handbooks-and-manuals/handbook (Accessed March 14, 2026).

[23] Booka E Kikuchi H Haneda R Soneda W Kawata S Murakami T . Impact of immune-related adverse events on nivolumab efficacy in patients with upper gastrointestinal cancer. In Vivo. (2021) 35:2321–6. doi: 10.21873/INVIVO.12506 34182512 PMC8286467

[24] Cao R Wu C Lv Y Xu S Li C Yin F . Correlation between immune-related adverse events and treatment efficacy of anti-PD1 immunotherapy in patients with esophageal squamous cell carcinoma. Oncol Lett. (2023) 25:55. doi: 10.3892/ol.2022.13641 36644142 PMC9827461

[25] Elghawy O Patel R Mullapudi A Kurian M Wang J Xu J . Immune-related adverse events not associated with survival in advanced or metastatic gastroesophageal cancers. J Chemother. (2026) 38:47–53. doi: 10.1080/1120009X.2024.2448644 39773445

[26] Hamai Y Ibuki Y Kurokawa T Hirohata R Ohsawa M Kitasaki N . Relationship of immune-related adverse events with tumor response and prognosis in esophageal squamous cell carcinoma following nivolumab monotherapy. Cancers (Basel). (2024) 16:3529. doi: 10.3390/cancers16203529 39456623 PMC11506036

[27] Hara Y Baba Y Toihata T Harada K Ogawa K Iwatsuki M . Immune-related adverse events and prognosis in patients with upper gastrointestinal cancer treated with nivolumab. J Gastrointest Oncol. (2022) 13:2779–88. doi: 10.21037/jgo-22-281 36636073 PMC9830324

[28] Hirasawa Y Kubota Y Mura E Suzuki R Tsurui T Iriguchi N . Maximum efficacy of immune checkpoint inhibitors occurs in esophageal cancer patients with a low neutrophil-to-lymphocyte ratio and good performance status prior to treatment. Anticancer Res. (2024) 44:3397–407. doi: 10.21873/anticanres.17160 39060084

[29] Ma H Zhang S Jiao P Ding H Wang F Zhao Y . Serum IL-6 predicts immunotherapy-related adverse and outcome in advanced gastric and esophageal cancer patients with anti-PD-1 treatment. Front Immunol. (2025) 16:1553882. doi: 10.3389/fimmu.2025.1553882 40519900 PMC12163003

[30] Nakada N Ando T Motoo I Kajiura S Fuchino M Sakumura M . MO5-1 Characteristics of immune-related adverse events in patients with advanced esophageal cancer treated with nivolumab. Ann Oncol. (2021) 32:S298. doi: 10.1016/j.annonc.2021.05.57142574925

[31] Natsuki S Lee S Kasashima H Miki Y Fukuoka T Yoshii M . Utility of assessing early tumor shrinkage as an efficacy predictor in patients with non-surgically indicated or recurrent esophageal cancer treated with nivolumab plus ipilimumab. Oncology. (2025) 103:167–78. doi: 10.1159/000540851 39208777

[32] Qin W Yang L Fan B Zou B Duan Y Li B . Association between immune-related adverse events and the efficacy of PD-1 inhibitors in advanced esophageal cancer. Front Immunol. (2022) 13:931429. doi: 10.3389/fimmu.2022.931429 36248782 PMC9554876

[33] Qiu Y Su G Zhang L Fan H Zhao H Wang C . Association between reactive cutaneous capillary endothelial proliferation and the efficacy of camrelizumab in esophageal cancer: a retrospective cohort study. J Thorac Dis. (2025) 17:2453–72. doi: 10.21037/jtd-2025-366 40400922 PMC12090165

[34] Wang R Ling Y Chen B Zhu Y Hu Y Liu M . Long-term survival and post-hoc analysis of toripalimab plus definitive chemoradiotherapy for oesophageal squamous cell carcinoma: insights from the EC-CRT-001 phase II trial. EClinicalMedicine. (2024) 75:102806. doi: 10.1016/j.eclinm.2024.102806 39281099 PMC11402426

[35] Zhang T Chen X Wu J Li J Qin Z Cao R . Impact of immune-related adverse events on treatment outcomes in advanced esophageal squamous cell carcinoma treated with immune checkpoint inhibitors. Front Immunol. (2026) 17:1741482. doi: 10.3389/fimmu.2026.1741482 41727433 PMC12916662

[36] Zhao YN Cong D Zhang W Jia Y Bai Y . Immune-related adverse events as independent prognostic factors for camrelizumab in patients with esophageal squamous cell carcinoma: a retrospective cohort study. J Gastrointest Oncol. (2023) 14:733–43. doi: 10.21037/jgo-23-75 37201088 PMC10186529

[37] Liang Y Xu H Liu F Li L Lin C Zhang Y . Immune-related adverse events and their effects on survival outcomes in patients with non-small cell lung cancer treated with immune checkpoint inhibitors: a systematic review and meta-analysis. Front Oncol. (2024) 14:1281645. doi: 10.3389/fonc.2024.1281645 38887231 PMC11180722

[38] Huang Y Ma W Wu D Lyu M Zheng Q Wang T . Prognostic relevance of immune-related adverse events in lung cancer patients undergoing immune checkpoint inhibitor therapy: a systematic review and meta-analysis. Transl Lung Cancer Res. (2024) 13:1559–84. doi: 10.21037/tlcr-24-299 39118883 PMC11304146

[39] Zhou X Yao Z Yang H Liang N Zhang X Zhang F . Are immune-related adverse events associated with the efficacy of immune checkpoint inhibitors in patients with cancer? A systematic review and meta-analysis. BMC Med. (2020) 18:87. doi: 10.1186/s12916-020-01549-2 32306958 PMC7169020

[40] Ibis B Aliazis K Cao C Yenyuwadee S Boussiotis VA . Immune-related adverse effects of checkpoint immunotherapy and implications for the treatment of patients with cancer and autoimmune diseases. Front Immunol. (2023) 14:1197364. doi: 10.3389/fimmu.2023.1197364 37342323 PMC10277501

[41] Du Y Wu W Chen M Dong Z Wang F . Cutaneous adverse events and cancer survival prognosis with immune checkpoint inhibitor treatment: a Systematic Review and Meta-Analysis. JAMA Dermatol. (2023) 159:1093–101. doi: 10.1001/jamadermatol.2023.3003 37672255 PMC10483383

[42] Teraoka S Fujimoto D Morimoto T Kawachi H Ito M Sato Y . Early immune-related adverse events and association with outcome in advanced non-small cell lung cancer patients treated with nivolumab: a prospective cohort study. J Thorac Oncol. (2017) 12:1798–805. doi: 10.1016/j.jtho.2017.08.022 28939128

[43] Hashimoto T Karashima T Kamada K Ogata K Ohta K Abe Y . Relationship between immune-related adverse events and treatment outcome of immune checkpoint inhibitors for advanced non-small cell lung cancer: a retrospective cohort study. J Thorac Dis. (2026) 18:89. doi: 10.21037/jtd-2025-1434 41816438 PMC12972837

[44] Hsiehchen D Naqash AR Espinoza M Von Itzstein MS Cortellini A Ricciuti B . Association between immune-related adverse event timing and treatment outcomes. Oncoimmunology. (2022) 11:2017162. doi: 10.1080/2162402X.2021.2017162 35003896 PMC8741287

[45] Fukushima T Kobayashi S Ueno M . The correlation between immune-related adverse events and efficacy of immune checkpoint inhibitors. Jpn J Clin Oncol. (2024) 54:949–58. doi: 10.1093/jjco/hyae067 38769817 PMC11374884

[46] Shimozaki K Sukawa Y Beppu N Kurihara I Suzuki S Mizuno R . Multiple immune-related adverse events and anti-tumor efficacy: real-world data on various solid tumors. Cancer Manag Res. (2020) 12:4585–93. doi: 10.2147/CMAR.S247554 32606951 PMC7305832

[47] Olsson Ladjevardi C Koliadi A Ryden V El-Naggar AI Digkas E Valachis A . Multiple immune-related adverse events secondary to checkpoint inhibitor therapy in patients with advanced cancer: association with treatment effectiveness. Front Oncol. (2024) 14:1399171. doi: 10.3389/fonc.2024.1399171 38988708 PMC11233440

[48] Burke KP Grebinoski S Sharpe AH Vignali DAA . Understanding adverse events of immunotherapy: a mechanistic perspective. J Exp Med. (2021) 218:e20192179. doi: 10.1084/jem.20192179 33601411 PMC7754677

[49] Sanlorenzo M Vujic I Daud A Algazi A Gubens M Luna SA . Pembrolizumab cutaneous adverse events and their association with disease progression. JAMA Dermatol. (2015) 151:1206–12. doi: 10.1001/jamadermatol.2015.1916 26222619 PMC5061067

[50] Hua C Boussemart L Mateus C Routier E Boutros C Cazenave H . Association of vitiligo with tumor response in patients with metastatic melanoma treated with pembrolizumab. JAMA Dermatol. (2016) 152:45–51. doi: 10.1001/jamadermatol.2015.2707 26501224

[51] Starace M Cedirian S Rapparini L Pileri A Carrera C Giavedoni P . Immune checkpoint inhibitor-induced vitiligo-like depigmentation. JAMA Dermatol. (2025) 161:216–8. doi: 10.1001/jamadermatol.2024.5136 39714856 PMC11840635

[52] Laubli H Koelzer VH Matter MS Herzig P Dolder Schlienger B Wiese MN . The T cell repertoire in tumors overlaps with pulmonary inflammatory lesions in patients treated with checkpoint inhibitors. Oncoimmunology. (2018) 7:e1386362. doi: 10.1080/2162402X.2017.1386362 29308309 PMC5749666

[53] Weinmann SC Pisetsky DS . Mechanisms of immune-related adverse events during the treatment of cancer with immune checkpoint inhibitors. Rheumatol (Oxford). (2019) 58:vii59–67. doi: 10.1093/rheumatology/kez308 31816080 PMC6900913

[54] Taylor J Gandhi A Gray E Zaenker P . Checkpoint inhibitor immune-related adverse events: a focused review on autoantibodies and B cells as biomarkers, advancements and future possibilities. Front Immunol. (2022) 13:991433. doi: 10.3389/fimmu.2022.991433 36713389 PMC9874109

[55] Toi Y Sugawara S Sugisaka J Ono H Kawashima Y Aiba T . Profiling preexisting antibodies in patients treated with anti-PD-1 therapy for advanced non-small cell lung cancer. JAMA Oncol. (2019) 5:376–83. doi: 10.1001/jamaoncol.2018.5860 30589930 PMC6439838

[56] de Moel EC Rozeman EA Kapiteijn EH Verdegaal EME Grummels A Bakker JA . Autoantibody development under treatment with immune-checkpoint inhibitors. Cancer Immunol Res. (2019) 7:6–11. doi: 10.1158/2326-6066.CIR-18-0245 30425107

[57] Blum SM Rouhani SJ Sullivan RJ . Effects of immune-related adverse events (irAEs) and their treatment on antitumor immune responses. Immunol Rev. (2023) 318:167–78. doi: 10.1111/imr.13262 37578634

[58] Dang QM Watanabe R Shiomi M Fukumoto K Nobashi TW Okano T . Rheumatic immune-related adverse events due to immune checkpoint inhibitors-a 2023 update. Int J Mol Sci. (2023) 24:5613. doi: 10.3390/ijms24065643 36982715 PMC10051463

[59] Schneider SM Fan C Wang Y Jenq RR Watowich SS . Microbiota and immune-related adverse events in cancer immunotherapy. Nat Rev Cancer. (2026) 26:480–96. doi: 10.1038/s41568-026-00921-3 41992000

[60] Casagrande S Sopetto GB Bertalot G Bortolotti R Racanelli V Caffo O . Immune-related adverse events due to cancer immunotherapy: immune mechanisms and clinical manifestations. Cancers (Basel). (2024) 16:1440. doi: 10.3390/cancers16071440 38611115 PMC11011060

[61] Teng YS Yu S . Molecular mechanisms of cutaneous immune-related adverse events (irAEs) induced by immune checkpoint inhibitors. Curr Oncol. (2023) 30:6805–19. doi: 10.3390/curroncol30070498 37504358 PMC10378098

[62] Matrai P Koi T Sipos Z Farkas N . Assessing the properties of the prediction interval in random-effects meta-analysis. Res Synth Methods. (2026) 17:517–37. doi: 10.1017/rsm.2025.10055 41992906 PMC13126221

[63] Yu Y Chen N Yu S Shen W Zhai W Li H . Association of immune-related adverse events and the efficacy of anti-PD-(L)1 monotherapy in non-small cell lung cancer: adjusting for immortal-time bias. Cancer Res Treat. (2024) 56:751–64. doi: 10.4143/crt.2023.1118 38186241 PMC11261188

[64] Cheung KM Tsui TYM Chow JCH . Guarantee-time bias in studies on the relationship between immune-related adverse events and antitumor activity. Cancer. (2022) 128:2549–50. doi: 10.1002/cncr.34244 35452127

