# Supplementary material for: Immune-related adverse events are associated with favorable clinical outcomes in esophageal cancer treated with immune checkpoint inhibitors: a systematic review and meta-analysis
Source: Front Oncol. 2026 Oct 6;16:1948772. doi: 10.3389/fonc.2026.1948772 (PMC13680211; doi:10.3389/fonc.2026.1948772)
Supplement: Supplementary file 1 [file SupplementaryFile1.docx]

**Catalogue:**

Text S1. Database Search Strategies.
Table S1. PRISMA 2020 Checklist. Provided as a separate supplementary file.
Table S2. Summary of study characteristics of all included studies.
Table S3. Variables included in the multivariate analysis reported by primary studies.
Table S4. Summary of immune-related adverse events reported in the included studies.

Table S5: Assessment of immortal-time bias in included studies.

Table S6: Results of the trim-and-fill analyses.
Figure S1. Risk of bias graph.

Figure S2: Additional exploratory subgroup analyses of the association between irAEs and survival outcomes.

Figure S3: Additional exploratory subgroup analyses of the association between irAEs and treatment response.
Figure S4. Sensitivity analysis of the impact of each individual study on the pooled effect.
Figure S5. Funnel plots of the pooled results.

**Database Search Strategies**

**Pubmed Search strategy**

#1

"Esophageal Neoplasms"[Mesh] OR "Esophageal Neoplasm"[Title/Abstract] OR "Neoplasm, Esophageal"[Title/Abstract] OR "Esophagus Neoplasm"[Title/Abstract] OR "Esophagus Neoplasms"[Title/Abstract] OR "Neoplasm, Esophagus"[Title/Abstract] OR "Neoplasms, Esophagus"[Title/Abstract] OR "Neoplasms, Esophageal"[Title/Abstract] OR "Cancer of Esophagus"[Title/Abstract] OR "Esophageal Cancer"[Title/Abstract] OR "Cancer, Esophageal"[Title/Abstract] OR "Cancers, Esophageal"[Title/Abstract] OR "Esophageal Cancers"[Title/Abstract] OR "Cancer of the Esophagus"[Title/Abstract] OR "Esophagus Cancer"[Title/Abstract] OR "Cancer, Esophagus"[Title/Abstract] OR "Cancers, Esophagus"[Title/Abstract] OR "Esophagus Cancers"[Title/Abstract]

#2

"Immune Checkpoint Inhibitors"[Mesh] OR "Checkpoint Inhibitors, Immune"[Title/Abstract] OR "Immune Checkpoint Blockers"[Title/Abstract] OR "Checkpoint Blockers, Immune"[Title/Abstract] OR "Immune Checkpoint Inhibitor"[Title/Abstract] OR "Checkpoint Inhibitor, Immune"[Title/Abstract] OR "CTLA-4 Inhibitors"[Title/Abstract] OR "CTLA 4 Inhibitors"[Title/Abstract] OR "Cytotoxic T-Lymphocyte-Associated Protein 4 Inhibitors"[Title/Abstract] OR "Cytotoxic T Lymphocyte Associated Protein 4 Inhibitors"[Title/Abstract] OR "Cytotoxic T-Lymphocyte-Associated Protein 4 Inhibitor"[Title/Abstract] OR "Cytotoxic T Lymphocyte Associated Protein 4 Inhibitor"[Title/Abstract] OR "CTLA-4 Inhibitor"[Title/Abstract] OR "CTLA 4 Inhibitor"[Title/Abstract] OR "PD-1 Inhibitors"[Title/Abstract] OR "PD 1 Inhibitors"[Title/Abstract] OR "Programmed Cell Death Protein 1 Inhibitor"[Title/Abstract] OR "Programmed Cell Death Protein 1 Inhibitors"[Title/Abstract] OR "PD-1 Inhibitor"[Title/Abstract] OR "Inhibitor, PD-1"[Title/Abstract] OR "PD 1 Inhibitor"[Title/Abstract] OR "Immune Checkpoint Blockade"[Title/Abstract] OR "Checkpoint Blockade, Immune"[Title/Abstract] OR "Immune Checkpoint Inhibition"[Title/Abstract] OR "Checkpoint Inhibition, Immune"[Title/Abstract] OR "PD-L1 Inhibitors"[Title/Abstract] OR "PD L1 Inhibitors"[Title/Abstract] OR "Programmed Death-Ligand 1 Inhibitors"[Title/Abstract] OR "Programmed Death Ligand 1 Inhibitors"[Title/Abstract] OR "PD-L1 Inhibitor"[Title/Abstract] OR "PD L1 Inhibitor"[Title/Abstract] OR "PD-1-PD-L1 Blockade"[Title/Abstract] OR "Blockade, PD-1-PD-L1"[Title/Abstract] OR "PD 1 PD L1 Blockade"[Title/Abstract] OR "Pembrolizumab"[Title/Abstract] OR "Nivolumab"[Title/Abstract] OR "Tislelizumab"[Title/Abstract] OR "Camrelizumab"[Title/Abstract] OR "Sintilimab"[Title/Abstract] OR "Toripalimab"[Title/Abstract] OR "Serplulimab"[Title/Abstract] OR "Sugemalimab"[Title/Abstract] OR "Ipilimumab"[Title/Abstract]

#3

"efficacy"[Title/Abstract] OR "prognosis"[Title/Abstract] OR "outcome"[Title/Abstract] OR "indicator"[Title/Abstract] OR "predict"[Title/Abstract] OR "predictor"[Title/Abstract] OR "response"[Title/Abstract] OR "association"[Title/Abstract] OR "benefit"[Title/Abstract]

#4

"immune-related adverse events"[Title/Abstract] OR "irAEs"[Title/Abstract] OR "adverse reactions"[Title/Abstract] OR "adverse effects"[Title/Abstract] OR "treatment-related adverse events"[Title/Abstract] OR "adverse events"[Title/Abstract]

#5

#1 and #2 and #3 and #4

**Web of Science Search strategy**

**#1**

(((((((((((((((((TS=(esophageal neoplasms)) OR TS=(Esophageal Neoplasm)) OR TS=(Neoplasm, Esophageal)) OR TS=(Esophagus Neoplasm)) OR TS=(Esophagus Neoplasms)) OR TS=(Neoplasm, Esophagus)) OR TS=(Neoplasms, Esophagus)) OR TS=(Neoplasms, Esophageal)) OR TS=(Cancer of Esophagus)) OR TS=(Esophageal Cancer)) OR TS=(Cancer, Esophageal)) OR TS=(Cancers, Esophageal)) OR TS=(Esophageal Cancers)) OR TS=(Cancer of the Esophagus)) OR TS=(Esophagus Cancer)) OR TS=(Cancer, Esophagus)) OR TS=(Cancers, Esophagus)) OR TS=(Esophagus Cancers)

#2

TS=(immune checkpoint inhibitor) OR TS=(Checkpoint Inhibitors, Immune) OR TS=(Immune Checkpoint Blockers) OR TS=(Checkpoint Blockers, Immune) OR TS=(Immune Checkpoint Inhibitor) OR TS=(Checkpoint Inhibitor, Immune) OR TS=(CTLA-4 Inhibitors) OR TS=(CTLA 4 Inhibitors) OR TS=(Cytotoxic T-Lymphocyte-Associated Protein 4 Inhibitors) OR TS=(Cytotoxic T Lymphocyte Associated Protein 4 Inhibitors) OR TS=(Cytotoxic T-Lymphocyte-Associated Protein 4 Inhibitor) OR TS=(Cytotoxic T Lymphocyte Associated Protein 4 Inhibitor) OR TS=(CTLA-4 Inhibitor) OR TS=(CTLA 4 Inhibitor) OR TS=(PD-1 Inhibitors) OR TS=(PD 1 Inhibitors) OR TS=(Programmed Cell Death Protein 1 Inhibitor) OR TS=(Programmed Cell Death Protein 1 Inhibitors) OR TS=(PD-1 Inhibitor) OR TS=(Inhibitor, PD-1) OR TS=(PD 1 Inhibitor) OR TS=(Immune Checkpoint Blockade) OR TS=(Checkpoint Blockade, Immune) OR TS=(Immune Checkpoint Inhibition) OR TS=(Checkpoint Inhibition, Immune) OR TS=(PD-L1 Inhibitors) OR TS=(PD L1 Inhibitors) OR TS=(Programmed Death-Ligand 1 Inhibitors) OR TS=(Programmed Death Ligand 1 Inhibitors) OR TS=(PD-L1 Inhibitor) OR TS=(PD L1 Inhibitor) OR TS=(PD-1-PD-L1 Blockade) OR TS=(Blockade, PD-1-PD-L1) OR TS=(PD 1 PD L1 Blockade) OR TS=(Pembrolizumab) OR TS=(Nivolumab) OR TS=(Tislelizumab) OR TS=(Camrelizumab) OR TS=(Sintilimab) OR TS=(Toripalimab) OR TS=(Serplulimab) OR TS=(Sugemalimab) OR TS=(Ipilimumab)

#3

TS=(efficacy) OR TS=(prognosis) OR TS=(outcome) OR TS=(indicator) OR TS=(predict) OR TS=(predictor) OR TS=(response) OR TS=(association) OR TS=(benefit)

#4

TS=(immune-related adverse events) OR TS=(irAEs) OR TS=(adverse reactions) OR TS=(adverse effects) OR TS=(treatment-related adverse events) OR TS=(adverse events)

#5

#1 and #2 and #3 and #4

**The Cochrane Library Search strategy**

**#1**

(MeSH descriptor: [Esophageal Neoplasms] explode all trees ) OR ( (Esophageal Neoplasm):ti,ab,kw OR (Neoplasm, Esophageal):ti,ab,kw OR (Esophagus Neoplasm):ti,ab,kw OR (Esophagus Neoplasms):ti,ab,kw OR (Neoplasm, Esophagus):ti,ab,kw ) OR ( (Neoplasms, Esophagus):ti,ab,kw OR (Neoplasms, Esophageal):ti,ab,kw OR (Cancer of Esophagus):ti,ab,kw OR (Esophageal Cancer):ti,ab,kw OR (Cancer, Esophageal):ti,ab,kw ) OR ( (Cancers, Esophageal):ti,ab,kw OR (Esophageal Cancers):ti,ab,kw OR (Cancer of the Esophagus):ti,ab,kw OR (Esophagus Cancer):ti,ab,kw OR (Cancer, Esophagus):ti,ab,kw ) OR ( (Cancers, Esophagus):ti,ab,kw OR (Esophagus Cancers):ti,ab,kw )

#2

(MeSH descriptor: [Immune Checkpoint Inhibitors] explode all trees ) OR ( (Checkpoint Inhibitors, Immune):ti,ab,kw OR (Immune Checkpoint Blockers):ti,ab,kw OR (Checkpoint Blockers, Immune):ti,ab,kw OR (Immune Checkpoint Inhibitor):ti,ab,kw OR (Checkpoint Inhibitor, Immune):ti,ab,kw ) OR ( (CTLA-4 Inhibitors):ti,ab,kw OR (CTLA 4 Inhibitors):ti,ab,kw OR (Cytotoxic T-Lymphocyte-Associated Protein 4 Inhibitors):ti,ab,kw OR (Cytotoxic T Lymphocyte Associated Protein 4 Inhibitors):ti,ab,kw OR (Cytotoxic T-Lymphocyte-Associated Protein 4 Inhibitor):ti,ab,kw ) OR ( (Cytotoxic T Lymphocyte Associated Protein 4 Inhibitor):ti,ab,kw OR (CTLA-4 Inhibitor):ti,ab,kw OR (CTLA 4 Inhibitor):ti,ab,kw OR (PD-1 Inhibitors):ti,ab,kw OR (PD 1 Inhibitors):ti,ab,kw ) OR ( (Programmed Cell Death Protein 1 Inhibitor):ti,ab,kw OR (Programmed Cell Death Protein 1 Inhibitors):ti,ab,kw OR (PD-1 Inhibitor):ti,ab,kw OR (Inhibitor, PD-1):ti,ab,kw OR (PD 1 Inhibitor):ti,ab,kw ) OR ( (Immune Checkpoint Blockade):ti,ab,kw OR (Checkpoint Blockade, Immune):ti,ab,kw OR (Immune Checkpoint Inhibition):ti,ab,kw OR (Checkpoint Inhibition, Immune):ti,ab,kw OR (PD-L1 Inhibitors):ti,ab,kw ) OR ( (PD L1 Inhibitors):ti,ab,kw OR (Programmed Death-Ligand 1 Inhibitors):ti,ab,kw OR (Programmed Death Ligand 1 Inhibitors):ti,ab,kw OR (PD-L1 Inhibitor):ti,ab,kw OR (PD L1 Inhibitor):ti,ab,kw ) OR ( (Pembrolizumab):ti,ab,kw OR (Nivolumab):ti,ab,kw OR (Tislelizumab):ti,ab,kw OR (Camrelizumab):ti,ab,kw OR (Sintilimab):ti,ab,kw ) OR ( (Toripalimab):ti,ab,kw OR (Serplulimab):ti,ab,kw OR (Sugemalimab):ti,ab,kw OR (Ipilimumab):ti,ab,kw )

#3

(immune-related adverse events):ti,ab,kw OR (irAEs):ti,ab,kw OR (adverse reactions):ti,ab,kw OR (adverse effects):ti,ab,kw OR (treatment-related adverse events):ti,ab,kw ) OR ( (adverse events):ti,ab,kw )

#4

(efficacy):ti,ab,kw OR (prognosis):ti,ab,kw OR (outcome):ti,ab,kw OR (indicator):ti,ab,kw OR (predict):ti,ab,kw ) OR ( (predictor):ti,ab,kw OR (response):ti,ab,kw OR (association):ti,ab,kw OR (benefit):ti,ab,kw )

#5

#1 and #2 and #3 and #4

**Embase Search strategy**

**#1**

'esophagus tumor'/exp OR 'esophageal neoplasm':ti,ab OR 'neoplasm, esophageal':ti,ab OR 'esophagus neoplasm':ti,ab OR 'esophagus neoplasms':ti,ab OR 'neoplasm, esophagus':ti,ab OR 'neoplasms, esophagus':ti,ab OR 'neoplasms, esophageal':ti,ab OR 'cancer of esophagus':ti,ab OR 'esophageal cancer':ti,ab OR 'cancer, esophageal':ti,ab OR 'cancers, esophageal':ti,ab OR 'esophageal cancers':ti,ab OR 'cancer of the esophagus':ti,ab OR 'esophagus cancer':ti,ab OR 'cancer, esophagus':ti,ab OR 'cancers, esophagus':ti,ab OR 'esophagus cancers':ti,ab

#2

'immune checkpoint inhibitor'/exp OR 'checkpoint inhibitors, immune':ti,ab OR 'immune checkpoint blockers':ti,ab OR 'checkpoint blockers, immune':ti,ab OR 'immune checkpoint inhibitor':ti,ab OR 'checkpoint inhibitor, immune':ti,ab OR 'ctla-4 inhibitors':ti,ab OR 'ctla 4 inhibitors':ti,ab OR 'cytotoxic t-lymphocyte-associated protein 4 inhibitors':ti,ab OR 'cytotoxic t lymphocyte associated protein 4 inhibitors':ti,ab OR 'cytotoxic t-lymphocyte-associated protein 4 inhibitor':ti,ab OR 'cytotoxic t lymphocyte associated protein 4 inhibitor':ti,ab OR 'ctla-4 inhibitor':ti,ab OR 'ctla 4 inhibitor':ti,ab OR 'pd-1 inhibitors':ti,ab OR 'pd 1 inhibitors':ti,ab OR 'programmed cell death protein 1 inhibitor':ti,ab OR 'programmed cell death protein 1 inhibitors':ti,ab OR 'pd-1 inhibitor':ti,ab OR 'inhibitor, pd-1':ti,ab OR 'pd 1 inhibitor':ti,ab OR 'immune checkpoint blockade':ti,ab OR 'checkpoint blockade, immune':ti,ab OR 'immune checkpoint inhibition':ti,ab OR 'checkpoint inhibition, immune':ti,ab OR 'pd-l1 inhibitors':ti,ab OR 'pd l1 inhibitors':ti,ab OR 'programmed death-ligand 1 inhibitors':ti,ab OR 'programmed death ligand 1 inhibitors':ti,ab OR 'pd-l1 inhibitor':ti,ab OR 'pd l1 inhibitor':ti,ab OR 'pd-1-pd-l1 blockade':ti,ab OR 'blockade, pd-1-pd-l1':ti,ab OR 'pd 1 pd l1 blockade':ti,ab OR 'pembrolizumab':ti,ab OR 'nivolumab':ti,ab OR 'tislelizumab':ti,ab OR 'camrelizumab':ti,ab OR 'sintilimab':ti,ab OR 'toripalimab':ti,ab OR 'serplulimab':ti,ab OR 'sugemalimab':ti,ab OR 'ipilimumab':ti,ab

#3

'immune-related adverse events':ti,ab OR 'iraes':ti,ab OR 'adverse reactions':ti,ab OR 'adverse effects':ti,ab OR 'treatment-related adverse events':ti,ab OR 'adverse events':ti,ab

#4

'efficacy':ab,ti OR 'prognosis':ab,ti OR 'outcome':ab,ti OR 'indicator':ab,ti OR 'predict':ab,ti OR 'predictor':ab,ti OR 'response':ab,ti OR 'association':ab,ti OR 'benefit':ab,ti

#5

#1 and #2 and #3 and #4

Table S2. Summary of study characteristics of all included studies.

| **Study** | **Version of CTCAE** | **Any-grade irAEs**  **(n/N)** | **Grade ≥3 irAEs**  **(n/N)** | **PFS HR (95%CI)** | **OS HR (95%CI)** | **ORR (%)**  **irAEs versus non-irAEs** | **DCR (%)**  **irAEs versus non-irAEs** | **NOS Quality Assessment Scale** |
| --- | --- | --- | --- | --- | --- | --- | --- | --- |
| Booka 2021 | v5.0 | 3/21 | NA | 0.067 (0.001–0.518) | 0.368 (0.046–2.967) | NA | NA | 8 |
| Cao 2023 | v4.03 | 39/78 | 9/78 | 0.281 (0.141–0.560) | 0.304 (0.151–0.611) | 15.4% vs 5.1% | 43.6% vs 15.4% | 9 |
| Hamai 2024 | v5.0 | 24/82 | 2/82 | NA | NA | 58.3% vs 6.9% | 83.3% vs 32.8% | 9 |
| Hara 2022 | v5.0 | 29/61 | NA | 0.351 (0.176–0.702) | 0.409 (0.196–0.854) | NA | NA | 8 |
| Hirasawa 2024 | v5.0 | 32/86 | 7/86 | 0.455 (0.282–0.735) | 0.343 (0.196–0.599) | NA | NA | 8 |
| Ma 2025 | v4.03 | 22/42 | NA | 1.421 (0.588–3.348) | 2.202 (0.421–11.526) | NA | NA | 8 |
| Nakada 2021 | NA | 9/34 | 0/34 | NA | NA | 62.5% vs 4.5% | 100.0% vs 59.1% | 7 |
| Qiu 2025 | v5.0 | 192/397 | 28/397 | 0.250 (0.180–0.340) | 0.670 (0.460–0.970) | NA | NA | 9 |
| Wang 2024 | v4.0 | 29/42 | 1/42 | 0.430 (0.190–0.930) | 0.590 (0.250–1.400) | NA | NA | 9 |
| Zhao 2023 | v4.0 | 81/136 | 15/136 | NA | 0.570 (0.420–0.770) | 39.5% vs 14.5% | 71.6% vs 47.3% | 9 |
| Qin 2022 | v5.0 | 143/295 | 33/295 | 0.655 (0.474–0.907) | 0.565 (0.397–0.806) | 37.8% vs 25.0% | 92.3% vs 83.6% | 9 |
| Natsuki 2025 | NA | 15/19 | NA | 0.781 (0.179–3.398) | NA | NA | NA | 8 |
| Elghawy 2026 | v5.0 | 11/40 | NA | 0.481 (0.163–1.424) | 0.565 (0.250–1.279) | NA | NA | 8 |
| Zhang 2026 | v5.0 | 79/213 | NA | NA | NA | 25.3% vs 20.9% | 96.2% vs 81.3% | 9 |

Note: NA, not reported; CTCAE, Common Terminology Criteria for Adverse Events; PFS, progression-free survival; OS, overall survival; ORR, objective response rate; DCR, disease control rate; HR, hazard ratio; CI, confidence intervals; irAEs, Immune-related adverse events; NOS, Newcastle-Ottawa Scale.

Table S3. Variables included in the multivariate analysis reported by primary studies.

| **Study** | **Variables** |
| --- | --- |
| Booka 2021 | NA |
| Cao 2023 | ECOG PS, Line of therapy, TNM stage, Combine therapy, postoperative recurrence, metastatic status |
| Hamai 2024 | NA |
| Hara 2022 | NA |
| Hirasawa 2024 | NA |
| Ma 2025 | NA |
| Nakada 2021 | NA |
| Qiu 2025 | age, sex, ECOG PS, family history of cancer, Pathological type, metastatic status, history of alcohol consumption, history of smoking, LDH level, number of camrelizumab treatment cycles completed, combination therapy, BMI, RCCEP, Albumin, PNI, NLR, PLR |
| Wang 2024 | NA |
| Zhao 2023 | age, sex, previous therapy, first-line platinum-based chemotherapy, metastatic status, ECOG PS, number of prior therapies, PD-L1 combined positive score, smoking status |
| Qin 2022 | ECOG PS, metastatic status, immunotherapy line, cycles, radiation, LDH level |
| Natsuki 2025 | NA |
| Elghawy 2026 | NA |
| Zhang 2026 | NA |

Note: NA, not reported; ECOG PS, Eastern Cooperative Oncology Group performance status; TNM, tumor node metastasis; BMI, body mass index; RCCEP, Reactive Cutaneous Capillary Endothelial Proliferation; PNI, prognostic nutritional index; NLR, neutrophil-to-lymphocyte ratio; PLR, platelet-to-lymphocyte ratio; DpR, depth of response; ETS, early tumor shrinkage; PD-L1, Programmed Death-Ligand 1.

Table S4. Summary of immune-related adverse events reported in the included studies.

| Study | All-grade irAEs (n, %) | Grade ≥3 irAEs (n, %) |
| --- | --- | --- |
| Booka 2021 | NA | NA |
| Cao 2023 | Hypothyroidism (12,15.38%), cutaneous capillary hyperplasia (15,19.23%), rash (8,10.26%), pneumonia (1,1.28%), adrenal insufficiency (2,2.56%), myositis/myocarditis (3,3.85%), AST/ALT/Bilirubin increased (2,2.56%), coliti (1,1.28%), hypophysitis (1,1.28%), Type 1 Diabetes (1,1.28%) | cutaneous capillary hyperplasia (2,2.56%), rash (2,2.56%), adrenal insufficiency (2,2.56%), myositis/myocarditis (1,1.28%), AST/ALT/Bilirubin increased (1,1.28%), Type 1 Diabetes (1,1.28%) |
| Hamai 2024 | Hypothyroidism (8,9.76%), rash (8,9.76%), pruritus (5,6.10%), pneumonitis (3,3.66%), diarrhea (3,3.66%), hepatic disorder (3,3.66%), adrenal insufficiency (1,1.22%), pancreatitis (1,1.22%), enterocolitis (1,1.22%), renal disorder (1,1.22%) | Pneumonitis (1,1.22%), adrenal insufficiency (1,1.22%) |
| Hara 2022 | NA | NA |
| Hirasawa 2024 | NA | Hepatitis (3,3.49%), pneumonitis (1,1.16%), hemophagocytic syndrome (1,1.16%), hypopituitarism (1,1.16%), rash (1,1.16%) |
| Ma 2025 | NA | NA |
| Nakada 2021 | skin rash, thyroiditis, hypothyroidism, colitis, cholangitis and thrombocytopenia | NA |
| Qiu 2025 | RCCEP (128,32.24%), skin rash/itch (17,4.28%), pneumonitis (13,3.27%), cardiovascular toxicities (7,1.76%), diarrhea/colitis (5,1.26%), infusion-related reactions (5,1.26%), elevated transaminase (4,1.01%), nephritis (4,1.01%), arthritis (4,1.01%), hyperglycemia (3,0.76%), myositis (3,0.76%), adrenal hypofunction (2,0.50%), neurovirulence (1,0.25%) | RCCEP (7,1.76%), skin rash/itch (4,1.01%), pneumonitis (4,1.01%),  cardiovascular toxicities (1,0.25%), diarrhea/colitis (2,0.50%), infusion-related reactions (1,0.25%), elevated transaminase (2,0.50%), nephritis (2,0.50%), myositis (1,0.25%), adrenal hypofunction (1,0.25%) |
| Wang 2024 | Hypothyroidism (13,30.95%), rash (12,28.57%), hyperlipidaemia (10,23.81%), fatigue (6,14.29%), oral mucositis (5,11.90%), hyperthyroidism (2,4.76%), hyperglycaemia (2,4.76%), neurotoxicity (2,4.76%), adrenal insufficiency (1,2.38%), diarrhoea (1,2.38%) | Hyperlipidaemia (1,2.38%) |
| Zhao 2023 | RCCEP (48, 35.29%), AST increased (12, 8.82%), diarrhea (13, 9.56%), hypothyroidism (12, 8.82%), fatigue (13, 9.56%), decreased appetite (9, 6.62%), nausea (8, 5.88%), vomiting (4, 2.94%), stomatitis (2, 1.47%), constipation (2, 1.47%), weight decreased (1, 0.74%), WBC count decreased (1, 0.74%), Neutrophil count decreased (1, 0.74%) | RCCEP (3, 2.20%), diarrhea (3, 2.20%),  hypothyroidism (2, 1.47%), fatigue (3, 2.20%), decreased appetite (2, 1.47%), nausea (1, 0.74%), vomiting (1, 0.74%) |
| Qin 2022 | anemia (49, 16.61%), hyper/hypothyroidism (45, 15.25%), pneumonitis (43, 14.58%), cardiovascular toxicities (35, 11.86%), fatigue (23, 7.80%), thrombocytopenia (22, 7.46%), fever (17, 5.76%), elevated transaminase (16, 5.42%), anorexia (15, 5.08%), reactive cutaneous capillary endothelial proliferation (13, 4.41%), nausea/vomiting (10, 3.39%), rash (5, 1.69%), pruritus (5, 1.69%), arthralgia/Myalgia (4, 1.36%), diarrhea/colitis (1, 0.34%) | pneumonitis (15, 5.08%), anemia (5, 1.69%), cardiovascular toxicities (3, 1.02%), elevated transaminase (3, 1.02%), hyper/hypothyroidism (2, 0.68%), thrombocytopenia (2, 0.68%), fatigue (1, 0.34%), reactive cutaneous capillary endothelial proliferation (1, 0.34%), rash (1, 0.34%) |
| Natsuki 2025 | rash, pruritus | NA |
| Elghawy 2026 | NA | NA |
| Zhang 2026 | endocrine (39, 18.31%), thyroiditis (29, 13.62%), adrenal (13, 6.10%), hypophysitis (5, 2.35%), myocarditis (5, 2.35%), diarrhea (3, 1.41%), hepatitis (10, 4.69%), dermatitis (13, 6.10%), pneumonitis (15, 7.04%), kidney injury (5, 2.35%), myositis (3, 1.41%) | NA |

Note: NA, not reported; ALT, Alanine Aminotransferase; AST, Aspartate Aminotransferase; RCCEP, Reactive Cutaneous Capillary Endothelial Proliferation; WBC, White Blood Cell.

Data for Nakada 2021 and Natsuki 2025 were reported descriptively only, and the corresponding numbers and percentages were not available due to missing data.

Table S5. Assessment of immortal-time bias in included studies.

| **Study** | **Time zero for survival** | **Reported irAEs timing / window** | **Survival analysis of irAEs** | **Adequately controls immortal-time bias** |
| --- | --- | --- | --- | --- |
| Booka 2021 | NA | irAEs assessed during treatment and through 28 days after treatment | Kaplan Meier and Log Rank Test | No |
| Cao 2023 | Start of immunotherapy | median onset 76 days (range 5-570 days) | Kaplan Meier, Log Rank Test and multivariable Cox regression | No |
| Hamai 2024 | Start of immunotherapy | median irAEs onset 60 days (range 14-427 days) | NA | No |
| Hara 2022 | NA | irAEs assessed during treatment until 1 month after the final dose or until next-line therapy | Kaplan Meier and Log Rank Test | No |
| Hirasawa 2024 | NA | NA | Kaplan Meier and Log Rank Test | No |
| Ma 2025 | NA | NA | Kaplan Meier and Log Rank Test | No |
| Nakada 2020 | NA | median irAEs onset 2.3 months (range 0.5-5.4 months) | NA | No |
| Qiu 2025 | Start of immunotherapy | most irAEs occurred within 3 months | NA | No |
| Wang 2024 | NA | NA | Kaplan Meier, Log Rank Test and multivariable Cox regression (PFS: no multivariate Cox regression analysis, OS: all of the above) | No |
| Zhao 2023 | Start of immunotherapy | irAEs occurring during the study or within 30 days after treatment; for irAEs of Grade 3 or higher, within 90 days | Kaplan Meier, Log Rank Test and multivariable Cox regression | No |
| Qin 2022 | NA | NA | Kaplan Meier, Log Rank Test and multivariable Cox regression | No |
| Natsuki 2025 | Start of immunotherapy | 13/15 irAEs cases occurred within 3 months | Kaplan Meier and Log Rank Test | No |
| Elghawy 2026 | NA | NA | Kaplan Meier and Log Rank Test | No |
| Zhang 2026 | Start of immunotherapy | median onset differs by organ system: dermatologic 1.90, hepatic 3.68, endocrine 4.17 and pulmonary 4.92 months | NA | No |

Note: NA, not reported.

**Note:** The assessment presented in this table pertains solely to whether immortal-time bias was appropriately addressed in comparisons of PFS and/or OS between patients with esophageal cancer who developed irAEs and those who did not, which is the comparison evaluated in the present meta-analysis. Analyses addressing other contrasts, such as irAEs onset time or specific irAEs subtypes, were not considered to have controlled immortal-time bias for this comparison.

Table S6. Results of the trim-and-fill analyses.

| **Outcome** | **Effect measure** | **Original pooled estimate (95% CI)** | **Imputed studies, n** | **Trim-and-fill adjusted estimate (95% CI)** | ***P* value** |
| --- | --- | --- | --- | --- | --- |
| PFS | HR | 0.45 (0.31-0.63) | 0 | 0.45 (0.31-0.63) | <0.001 |
| OS | HR | 0.54 (0.46-0.64) | 1 | 0.56 (0.48-0.66) | <0.001 |
| ORR | OR | 4.00 (1.58-10.12) | 3 | 1.75 (0.55-5.55) | 0.339 |
| DCR | OR | 3.85 (2.40-6.20) | 3 | 2.89 (1.71-4.90) | <0.001 |

Note: CI, confidence interval; HR, hazard ratio; OR, odds ratio; PFS, progression-free survival; OS, overall survival; ORR, objective response rate; DCR, disease control rate. Imputed studies represent hypothetical missing studies estimated using the trim-and-fill method. *P* values correspond to the trim-and-fill adjusted pooled estimates.

Figure S1. Risk of bias graph (Bias from eight key sources assessed with the NOS criteria).

Figure S2. Additional exploratory subgroup analyses of the association between irAEs and survival outcomes. (A) PFS; (B) OS. Estimates from a single study are shown as gray markers with dashed 95% CI lines and interpreted descriptively. irAEs, immune-related adverse events; PFS, progression-free survival; OS, overall survival; HR, hazard ratio; CI, confidence interval.

Figure S3. Additional exploratory subgroup analyses of the association between irAEs and treatment response. (A) ORR; (B) DCR. Estimates from a single study are shown as gray markers with dashed 95% CI lines and interpreted descriptively. irAEs, immune-related adverse events; ORR, objective response rate; DCR, disease control rate; OR, odds ratio; CI, confidence interval.

Figure S4. Sensitivity analysis of the impact of each individual study on the pooled effect. (A) PFS; (B) OS, (C) ORR; (D) DCR. PFS, progression-free survival; OS, overall survival; ORR, objective response rate; DCR, disease control rate.

Figure S5. Funnel plots of the pooled results. (A) PFS; (B) OS; (C) ORR; (D) DCR. PFS, progression-free survival; OS, overall survival; ORR, objective response rate; DCR, disease control rate.
